# The ingredients of a great scientific lecture

**DOI:** 10.1038/s44319-025-00382-z

**Published:** 2025-01-31

**Authors:** David R Smith

**Affiliations:** Department of Biology, University of Western Ontario, 1151 Richmond Street London, Ontario, N6A 5B7 Canada

**Keywords:** Careers, History & Philosophy of Science

## Abstract

Scientific lectures are often dense, boring, and hard to understand. We need to do better at presenting our data in an accessible and engaging manner.

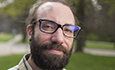

You all know that feeling: the presentation starts, the topic is interesting, the introduction piques your attention, and it all seems to make sense. Then, on the tenth graph, the twentieth bullet point, the fifth scatter blot, your eyes glaze over. You no longer have any idea what you are looking at. You shake your head, search your scientific soul, but you come up empty. You realize you have no bloody clue what this person is talking about and pray to the communication gods that they do not go over time. But they inevitably do!

Let’s be honest, many scientific lectures are boring, and some are downright dreadful. Having now spent over a thousand hours sitting in conferences, I can say with some conviction that, at best, one in ten is excellent. The other nine are another story. I am embarrassed to admit that I take away very little from most of the conference talks I attend. The same goes for university seminars and keynote speeches. Is it me? Am I just too slow to keep up with a typical scientific lecture within my field or are most scientists bad at presenting their data in an accessible and entertaining manner that will stick to my memory after the final slide?

Terrible talks do not discriminate. They are as likely to occur to a graduate student as they are to a seasoned pro wielding a large H-index. I have traveled across oceans to far-flung meetings to see my favorite scientists speak only to find myself disappointed and wishing I’d stayed home and watched a TED Talk instead. Why did they cram fifty slides into a fifteen-minute window? Why did the presentation contain more text than a mid-sized novella? Why in Picasso’s name did they pick pink as the background colour? Why are they trying to sell me 8-years’ worth of unrelenting data when all I want is a straightforward, bite-sized summary? I always know there’s going to be trouble when a speaker starts by saying: “Today I’m going to tell you four different stories…” Have mercy! I have a hard enough time handling half a story when your figures are that complex.

When I was an impressionable postdoc, I had the privilege of taking an invited speaker out to lunch before his lecture. The stakes were high: it was part of a job application for a chaired position. I think I felt more nervous than the speaker knowing I had to get him fed, out of the restaurant and to the seminar room with ample time to prepare. With our meals finished and the clock ticking he said: “Dave, before we head out, I’m just going to have a quick espresso.” I did the math. It would be cutting it close, but we could swing it. Then something happened that shook me to my core. The coffee arrived and the speaker took out his laptop and opened a PowerPoint file. OK, I thought, he is just getting ready for the talk. But then he opened two other PowerPoint presentations and started copying and pasting large numbers of slides into the first file all while simultaneously talking to me about the real-estate market in San Diego. My anxiety erupted. This can not be happening. We have exactly 24 min before “go time” and this guy’s willy-nilly throwing together a keynote lecture. Of course, the presentation was as dense and disjointed as a draft genome assembly from the early 2000s. But his publication record was on his side, so he was offered the position. The thing is, this individual was brilliant, engaging and doing exciting science. He had all the ingredients for delivering a great talk. Except the most important one: giving a damn.

I can already hear the critics shouting at their screens. “Alright, Mr. Critical, let’s watch you give a talk and see if we are still smiling by the end of it.” I am not claiming to be better than anyone else, nor is my scientific output anything to brag about. But I do give a damn about giving a good talk. As a PhD student I had a mentor, named Bob, who cared deeply about effective communication, and he instilled in me a similar passion. He would encourage me to practice my talks repeatedly: “Smitty,” he would say, “it’s too technical, too impenetrable. You need to dumb it down!” He would always preach about the four main rules for a strong presentation: keep it short, keep it simple, tell a story (with emphasis on the “a”), and engage your audience.

Bob’s true test of a perfectly structured presentation was that the speaker should be able to ditch the PowerPoint slides and successfully deliver the same message using only the blackboard. In fact, he would hold that most talks would indeed be better when boiled down to the bare basics: chalk board, presenter, ideas. I’m not suggesting you arrive at your next invited seminar with only an erasable marker in your hand. But when you do sit down to draft your next presentation, try thinking about how you could build it in a way that would work without PowerPoint as its foundation.

Now, I am not as cynical as I may seem. I believe that everyone, no matter their education, has it in them to speak compellingly about something they are passionate about. And for all my whining and complaining, I have witnessed some truly marvelous scientific talks throughout my life. The kind of talks where you walk away saying: “*This* is why I am a scientist!” In the first year of my Master’s, I attended a departmental seminar by a well-known biologist. Although the topic was about evolutionary genetics and the Tree of Life, the speaker mainly used images of famous classical paintings to make his points. It was magical, captivating and changed the way I understood effective scientific communication. That presentation is one of the reasons I became an evolutionary geneticist.

If you see me in the audience at your next talk, do not be nervous. Just remember Bob’s four rules. And know that if your slides do not load or AV cords will not connect, I will be the first one to step up and hand you a fresh piece of chalk.

## Supplementary information

Peer Review File

