# Supplementary material for: The ingredients of a great scientific lecture
Source: EMBO Rep. 2025 Jan 31;26(4):909–10. doi: 10.1038/s44319-025-00382-z (PMC11850849; doi:10.1038/s44319-025-00382-z)
Supplement: Supplementary file 1 — Peer Review File [file 44319_2025_382_MOESM1_ESM.pdf]

EMBO reports

The ingredients of a great scientific lecture

David Smith

Corresponding author(s): David Smith (dsmit242@uwo.ca)

Review Timeline:	Submission Date:	21st Jan 25
	Accepted:	21st Jan 25

Editor: Holger Breithaupt

Transaction Report: This article type is not peer reviewed.

(Note: With the exception of the correction of typographical or spelling errors that could be a source of ambiguity, letters and reports are not edited. Depending on transfer agreements, referee reports obtained elsewhere may or may not be included in this compilation. Referee reports are anonymous unless the Referee chooses to sign their reports.)
